# Cationic LNP-formulated mRNA expressing Tie2-agonist in the lung endothelium prevents pulmonary vascular leakage

**DOI:** 10.1016/j.omtn.2023.102068

**Published:** 2023-10-29

**Authors:** Katrin Radloff, Birgitt Gutbier, Charlotte Maeve Dunne, Hanieh Moradian, Marko Schwestka, Manfred Gossen, Katharina Ahrens, Laura Kneller, Yadong Wang, Akanksha Moga, Leonidas Gkionis, Oliver Keil, Volker Fehring, Daniel Tondera, Klaus Giese, Ansgar Santel, Jörg Kaufmann, Martin Witzenrath

**Affiliations:** 1 Pantherna Therapeutics GmbH, 16761 Hennigsdorf, Germany; 2 Charité - Universitätsmedizin Berlin, Corporate Member of Freie Universität Berlin and Humboldt-Universität zu Berlin, Department of Infectious Diseases, Respiratory Medicine, and Critical Care, 10117 Berlin, Germany; 3 Institute of Active Polymers, Helmholtz-Zentrum Hereon, 14513 Teltow, Germany; 4 Berlin-Brandenburg Center for Regenerative Therapies (BCRT) Charité Campus Virchow Klinikum, 13353 Berlin, Germany

**Keywords:** MT: Oligonucleotides: Therapies and Applications, cationic lipid nanoparticles, mRNA, tissue-specific targeting of the lung vasculature, therapeutic modulation of gene expression, endothelium, acute respiratory distress syndrome, ARDS, edema

## Abstract

Dysfunction of endothelial cells (ECs) lining the inner surface of blood vessels are causative for a number of diseases. Hence, the ability to therapeutically modulate gene expression within ECs is of high therapeutic value in treating diseases such as those associated with lung edema. mRNAs formulated with lipid nanoparticles (LNPs) have emerged as a new drug modality to induce transient protein expression for modulating disease-relevant signal transduction pathways. In the study presented here, we tested the effect of a novel synthetic, nucleoside-modified mRNA encoding COMP-Ang1 (mRNA-76) formulated into a cationic LNP on attenuating inflammation-induced vascular leakage. After intravenous injection, the respective mRNA was found to be delivered almost exclusively to the ECs of the lung, while sparing other vascular beds and bypassing the liver. The mode of action of mRNA-76, such as its activation of the Tie2 signal transduction pathway, was tested by pharmacological studies *in vitro* and *in vivo* in respective mouse models. mRNA-76 was found to prevent lung vascular leakage/lung edema as well as neutrophil infiltration in a lipopolysaccharide-challenging model.

## Graphical abstract

**Figure:**
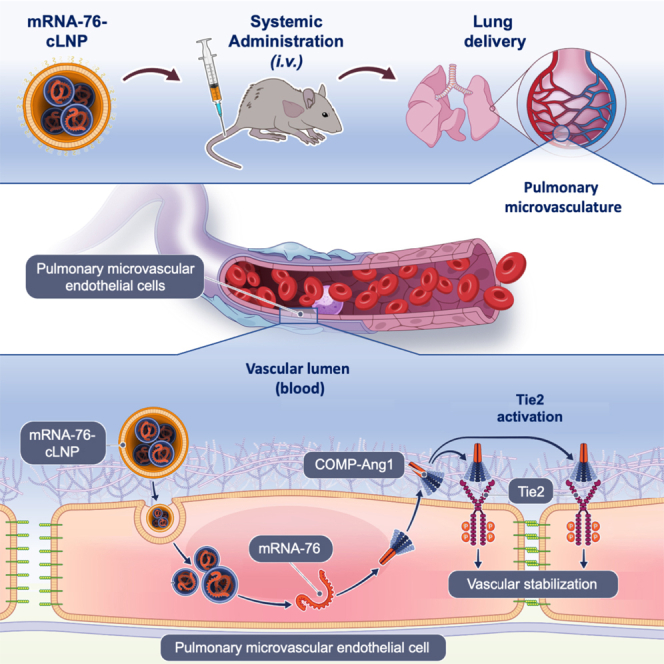

## Teaser

Kaufmann and colleagues reported selective mRNA delivery by novel cationic lipid nanoparticles (LNPs) to pulmonary endothelium. Lung edema occurs via the loss of the barrier function of blood capillaries, which can be restored by positioning a Tie2 agonist at the lung endothelium using mRNA-LNP, offering an mRNA therapy for diseases with underlying vascular leakage.

## Introduction

The ability to modulate gene expression in endothelial cells (ECs) by localized delivery of nucleic acids has been a therapeutic goal for various disease challenges such as stroke, heart disease, diabetes, vascular disease, and severe infectious diseases and their treatment sequelae.^1^ For this reason, delivery of therapeutic mRNA specifically to the ECs of specific organs and tissues has emerged as a new category of promising therapeutic agents. In nonviral gene delivery, a major advantage of mRNA compared to DNA cargoes is that mRNA does not require delivery into the cell nucleus. As a result, mRNA can achieve robust expression even of those proteins that are difficult to produce, especially in challenging cell types. However, for successful *in vivo* function, mRNA requires safe, effective, and stable delivery systems, which protect the nucleic acid from degradation and which allow for intracellular delivery of anionic nucleic acids, the latter known not to readily traverse the hydrophobic cell membrane lipid bilayer. A very potent delivery system for nucleic acids are lipid nanoparticles (LNPs), which were first approved as a small interfering RNA (siRNA) drug delivery vehicle in 2018 for the treatment of hereditary transthyretin-mediated amyloidosis.^2^ LNPs have since then successfully entered the clinic for the delivery of mRNA, as demonstrated by the US Food and Drug Administration approval of several other drugs, among them the coronavirus disease 2019 vaccines BNT162b2 and mRNA-1273 in 2021 and 2022.^2^^,^^3^^,^^4^

However, the targeting scope of systemically administered LNPs coformulated with siRNA or mRNA has been expanded beyond their traditional tropism for hepatic and splenic tissues. Recognizing the vast therapeutic potential of mRNA-based interventions, researchers have pioneered strategies to guide mRNA delivery to extrahepatic sites. There has been a pronounced emphasis on devising RNA delivery systems that can selectively target and transfect ECs throughout the vasculature, with a heightened focus on the ECs of the pulmonary vasculature. A strategy that has demonstrated efficacy uses permanently charged cationic lipids and/or cationic lipidoids in the construction of nanoparticles tailored for EC targeting. Leveraging this approach, delivery platforms have been engineered that exhibit proficiency in transfecting ECs across diverse organs, encompassing the lung, heart, and liver.^5^^,^^6^^,^^7^ In addition, specialized delivery systems have been developed with a specific affinity for the lung tissue and the ECs of the pulmonary vasculature.^8^^,^^9^^,^^10^^,^^11^

In recent advancements, the selective organ targeting (SORT) methodology has been introduced to refine neutral ionizable LNPs, originally designed for hepatocyte delivery. The biodistribution of these LNPs is modulated through the integration of an additional component, called the SORT lipid. By incorporating such specific lipids, either cationic or anionic in nature, it becomes feasible to meticulously adjust the *in vivo* mRNA delivery profile, thereby reducing hepatic sequestration and enhancing targeted mRNA delivery to pulmonary and splenic tissues.^12^^,^^13^ At present, it seems to remain unclear whether the use of traditional cationic formulations or the use of cationic SORT formulations offers an (therapeutic) advantage for the transport of mRNA to ECs of the vasculature. One could also argue that the SORT formulations merely represent a specific case of the traditional cationic formulations because they consist of a more complex lipid system but exhibit very similar physicochemical properties. However, in terms of chemistry, manufacturing and controls considerations, the SORT-LNP with its five-lipid moiety may be more challenging compared to the three-lipid-containing traditional cationic LNP (cLNP).

ECs line the inner surface of blood vessels, and pulmonary ECs play a pivotal role, not only in optimizing gas exchange and in controlling barrier integrity and function but also in regulating pulmonary vascular tone, owing to their strategic position at the interface between the bloodstream and lung tissue. Alterations in the pulmonary endothelium as a vital part of the respiratory system play a central role in the pathogenesis of several common and rare chronic and acute lung diseases.^14^^,^^15^^,^^16^ One of the main characteristics of pulmonary endothelial alteration (or dysfunction) is an increase in its permeability, which can lead to vascular leakage and edema formation. In molecular terms, the Ang/Tie-2 receptor tyrosine kinase signaling pathway has been shown to regulate vascular homeostasis and to control vessel permeability, inflammation, and angiogenic responses.^17^ Agonistic Ang1 and antagonistic Ang2 are both ligands for the receptor tyrosine kinase Tie2. In particular, the inactivation of Tie2 signaling due to an inflammatory increase in Ang2 secretion is considered to be one of the major molecular mechanisms for the impairment of barrier function in the lung vasculature. Conversely, the activation of Tie2 signaling by Ang1 restores endothelial barrier function and prevents further vascular leakage. This unique function of the Ang-Tie2 pathway in vascular stabilization renders this pathway as an attractive and validated target in conditions with an impaired endothelial barrier.^18^ Highly oligomeric, recombinant Ang1 and its chimeric hyperactive derivates COMP-Ang1 and CMP-Ang1 have previously been shown to activate Tie2 signaling but are difficult to produce, purify, and store in a stable and active form.^19^^,^^20^ Hence, recombinant production of Ang1 multimers under Good Manufacturing Practices conditions has not been possible for clinical use thus far. In addition, full-length Ang1 was previously demonstrated to be incorporated into the extracellular matrix (ECM) via its linker peptide region and to subsequently undergo rapid serum clearance.^20^^,^^21^^,^^22^

Here, we introduce a novel cLNP nucleic acid delivery system for transporting mRNAs selectively and almost exclusively into capillary ECs of the lung vasculature. We demonstrate that our mRNA-based approach of directed expression of a novel synthetic, nucleoside-modified mRNA-76 encoding a hyperactive human Ang1-derived fusion protein (hCOMP-Ang1) in pulmonary ECs transiently activates the Tie2 pathway *in vitro* and *in vivo*. We subsequently demonstrate the effect of delivering COMP-Ang1-encoding mRNA formulated into cLNPs on attenuating inflammation-induced vascular leakage in the lung endothelium.

## Results

### cLNP-mediated mRNA delivery into specific cell types of murine lung

**Figure 1:**
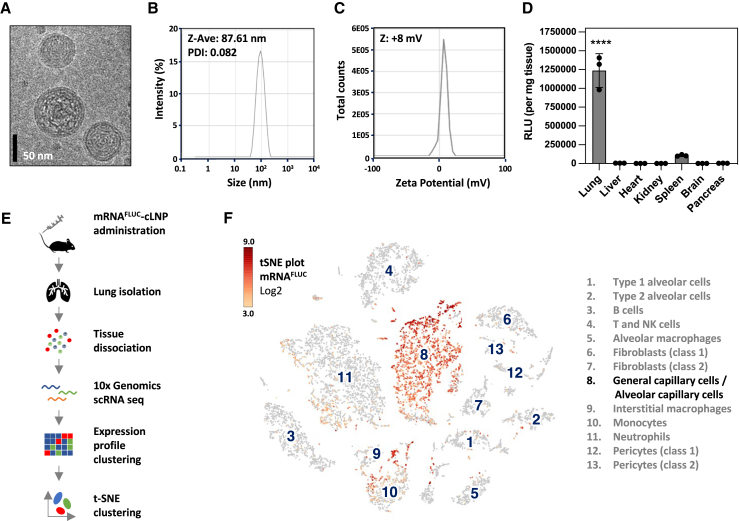
cLNP-mediated delivery leads to mRNA uptake predominantly in murine pulmonary capillary ECs (A) Cryoelectron microscopy, (B) dynamic light scattering analysis of particle size (Z-Ave), PDI, and (C) zeta potential of cLNP. (D) Luciferase assay of indicated tissue samples from mice 6 h after i.v. injection of 1.5 mg/kg of cLNP containing mRNA encoding for nonsecreted Firefly luciferase (mRNA^FLUC^) shown by relative light units (RLUs) per milligram of tissue. Data are represented as mean ± SEM; p < 0.0001. (E) Experimental workflow of single-cell RNA sequencing (scRNA seq) analysis of murine lung cells and results of loupe-based t-SNE clustering of murine lung cell types clustered by single-cell transcriptional analysis, showing a total of 13 distinct cell-type clusters as indicated by numbers. (F) t-SNE plots showing Log2 levels for Luciferase-encoding mRNA^FLUC^. NK, natural killer.

To introduce our delivery system, we formulated reporter mRNA (nonsecreted Firefly luciferase, mRNA^FLUC^) within our cLNPs and administered them by intravenous (i.v.) tail vein injection into mice. The physical characteristics of the prepared mRNA^FLUC^-cLNP formulations including cryoelectron microscopy analysis and measurement of particle size and surface charge are shown in Figures 1A–1C. The luciferase assay (Figure 1D) of the indicated tissue samples from mice 6 h after intravenous injection of 1.5 mg/kg of cLNP containing mRNA^FLUC^ illustrates expression predominantly in the lung, with minor amounts of expression also occurring in the spleen. Using single-cell RNA sequencing we were able to demonstrate that intravenous delivery of synthetic mRNA^FLUC^-cLNP leads to the uptake of respective payload almost exclusively in murine pulmonary capillary ECs. Figure 1E depicts the experimental workflow of single-cell RNA sequencing analysis of the murine lung, and Figure 1F depicts the results of loupe-based t-distributed stochastic neighbor embedding (t-SNE) clustering of murine lung cell types. A total of 13 distinct cell type clusters as indicated by numbers were identified, and the localization of mRNA^FLUC^ expression was found to occur predominantly in the general and alveolar capillary cells of the murine lung tissue, with low levels of expression also occurring in respective monocytes.

### mRNA-directed expression, secretion, and multimerization of Ang-1, COMP-Ang-1, and CMP-Ang-1 *in vitro*

**Figure 2:**
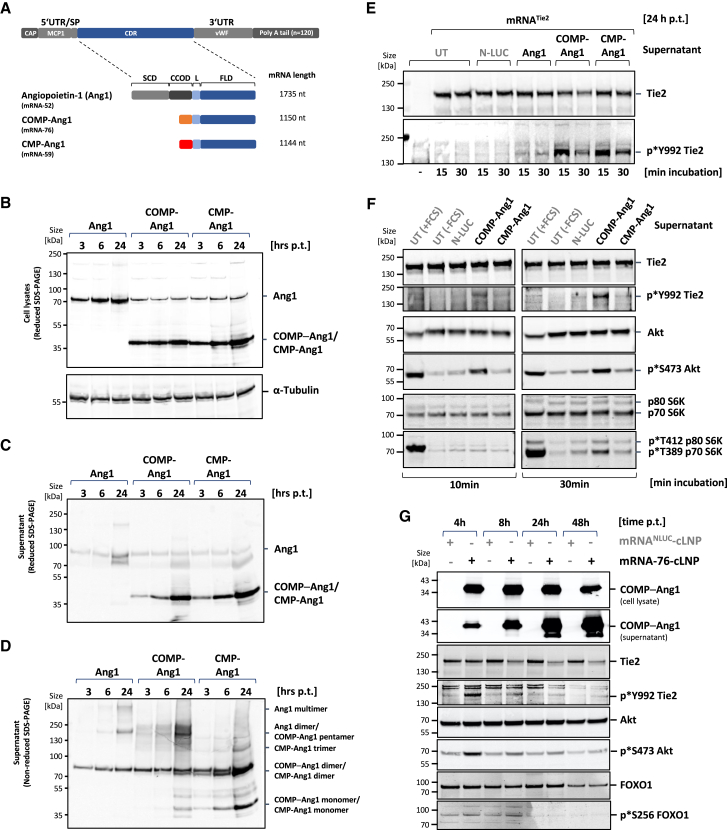
Expression and Tie2 activation by mRNA-directed expression of human COMP-Ang-1 *in vitro* (A) General structure of mRNA with signal peptide (SP) sequence flanked by designated 5′ and 3′ UTR regions plus defined polyA-tail. Schematic representation of protein domain structure of the natural protein Ang1 and the derived engineered humanized fusion proteins COMP-Ang1 and CMP-Ang1. Protein domains of human Ang1: SCD, super-clustering domain; CCOD, coiled-coil domain; L, linker; FLD, Fibrinogen-related domain; the multimerization domains of COMP (orange) and of CMP protein (red) are indicated. (B) Expression, (C) secretion, and (D) multimerization of full-length Ang1 and Ang1 derivates in HeLa cells. Cells were transfected for 2 h with mRNA-52 encoding Ang1, mRNA-76 encoding COMP-Ang1, or mRNA-59 encoding CMP-Ang1. Cell lysates (B) and supernatants (C, D) were harvested at indicated time points posttransfection (p.t.) and analyzed by Western blotting using Ang1-specific antibody. (E) Immunoblot of Tie2 expression and Tie2 phosphorylation from mRNA^Tie2^–transfected HeLa cells after 15 or 30 min incubation with supernatant from HeLa cells expressing secreted Nano-Luciferase (N-LUC), Ang1, COMP-Ang1, and CMP-Ang-1 (see C, D). (F) Activation of Tie2 pathway in serum-starved primary HPMECs by COMP-Ang1 and by CMP-Ang1 containing supernatant from mRNA-76 or mRNA-59 transfected HeLa cells. Tie2-pathway activation is demonstrated by Tie2 phosphorylation status and by the presence of phosphorylation at the downstream effectors Akt and S6K. (G) Tie2-pathway activation after transfection of LNP-formulated, COMP-Ang1-encoding mRNA-76 in serum-starved HPMECs. COMP-Ang1 expression, secretion, Tie2, Akt, and FOXO1 phosphorylation is shown by immunoblot on cell lysates at different time points p.t. FCS, fetal calf serum; Luc, control Nano-Luciferase-encoding mRNA; nt, nucleotides; UT, untreated.

Subsequently, the expression of the coding sequences of the wild-type (WT) human Ang1 and of engineered fusion proteins COMP-Ang1 and CMP-Ang1 flanked by heterologous untranslated regulatory sequences was tested in cell culture systems to identify a potent mRNA construct for Tie2 activation (Figure 2). COMP-Ang1 and CMP-Ang1 recombinant fusion proteins have been previously described containing the rat homolog of COMP- or human CMP-multimerization domain as a potent Tie2 agonist in *in vitro* assays and in mouse models. The expression of coding sequences of the WT human Ang1 and of the respective fusion proteins COMP-Ang1 and CMP-Ang1 flanked by heterologous untranslated regulatory sequences was tested in cell culture systems for the identification of a potent mRNA construct for Tie2 activation. WT human Ang1 mRNA with heterologous UTRs served as a control next to either a construct with an open reading frame (ORF) for a COMP-Ang1 fusion protein or to a construct containing an ORF with a CMP-Ang1 fusion protein (Figure 2A) directing the transient expression of human Ang1 and COMP-Ang1 and CMP-Ang1 fusion proteins *in vitro*. Subsequently, expression (Figure 2B), secretion (Figure 2C), and multimerization (Figure 2D) of WT-Ang1 and Ang1 derivatives was demonstrated. To this end, mRNAs encoding WT human Ang1 and fusion proteins COMP-Ang1 and CMP-Ang1 were transfected into HeLa cells, and cell culture supernatants were collected 3, 6, and 24 h posttransfection. Corresponding whole-cell lysates were prepared. The supernatants and cell lysates were subsequently analyzed by immunoblotting using an Ang1-specific antibody. A strong protein expression signal was detected for all three constructs in cell lysates (Figure 2B). Equivalently high protein levels of COMP-Ang1 and CMP-Ang1 were detected in the supernatant of the cell culture, indicating efficient secretion of the expressed proteins, whereas the level of soluble WT-Ang1 was strongly reduced (Figure 2C). This observation is consistent with *in vivo* data by Xu and Yu, demonstrating that full-length Ang1, unlike Ang2, is incorporated efficiently into the ECM via its linker peptide region, thereby explaining its rapid serum clearance.^23^ The multimerization potential of the expressed Ang1 derivates was analyzed by using nonreducing PAGE on the supernatant. COMP-Ang1 fusion protein was found to be capable of forming stable pentamers, contrasting WT-Ang1, shown to form dimers and multimers, and CMP-Ang1, present primarily in the form of monomers and dimers (Figure 2D).

### Activation of Tie2 by COMP-Ang1 and CMP-Ang1

COMP-Ang1 encoded by mRNA-76 was found in our subsequent experiments to activate Tie2 in a paracrine manner *in vitro*. Tie2^−^ HeLa cells were first transfected for 24 h with mRNA encoding for Tie2 receptor using Lipofectamine MessengerMax. Subsequently, cells were incubated for 15 or 30 min with supernatants from HeLa cells previously transfected for 24 h with mRNA encoding the different Ang1 derivatives, or with mRNA encoding for secreted NanoLuc-luciferase (mRNA^NLUC^). Then, Tie2-transfected HeLa cells were lysed, and protein levels analyzed by Western blotting. Both supernatants of Ang1 derivatives showed a significantly stronger activation via phospho-Y992 Tie2 antibody binding to the Tie2 receptor (Figure 2E) than the luciferase control and WT-Ang1 proteins, suggesting a superior paracrine activity of COMP-Ang1 and CMP-Ang1 as compared to WT-Ang1. The ability of different Ang1 derivatives to induce paracrine activation of the Tie2 pathway in human primary ECs was then investigated in a second set of experiments. To this end, starved primary human primary microvascular ECs (HPMECs) were incubated for 10 and 30 min with supernatants from HeLa cells previously transfected in serum-free medium for 24 h with mRNAs and encoding the different Ang1 derivatives or with NanoLuc-encoding mRNA as a control. HPMEC cells were lysed after 10 and 30 min and the phosphorylation status of Tie2, Akt, and S6K analyzed by Western blotting and compared to lysates from starved cells or from cells stimulated for 10 and 30 min with fetal calf serum (FCS) (Figure 2F). The strongest downstream activation of Akt and S6K was seen after FCS treatment of starved cells, thus corroborating the expected broad growth factor response induced by FCS. Cells treated with supernatant from HeLa cells transfected with mRNA-76 encoding COMP-Ang1 also displayed robust phosphorylation of Akt and of S6K, but additionally exhibited autophosphorylation of Tie2, indicative of a more specific pathway activation. The paracrine effect of HeLa conditional medium–derived COMP-Ang1 resulted in a better Tie2 pathway activation as compared to CMP-Ang1, leading to the selection of mRNA-76 for all subsequent experiments. mRNA-76 encoding COMP-Ang1 formulated with cLNP was directly transfected into HPMEC and secretion, and Tie2 signaling was analyzed for demonstrating an increased autocrine Tie2 activation. Strong COMP-Ang1 expression was observed in cell lysates and in the supernatant of the respective culture, indicating efficient secretion (Figure 2G). Lysate analysis of serum-starved HPMEC transfected with mRNA-76 revealed phosphorylation of Tie2, Akt, and FOXO1, thereby indicating functional Tie2 pathway activation at 4 and 8 h posttransfection, as well as rapid onset of activation. Waning of Tie2 receptor activation and reduction of Tie2 receptor protein levels were observed at 24 and 48 h post-transfection, consistent with previous reports describing a ligand-dependent Tie2 internalization and degradation,^24^^,^^25^^,^^26^ as well as reduced expression of Tie2 mRNA upon Ang1 treatment.^27^

### COMP-Ang1 expression stabilizes endothelial barrier function *in vitro*

**Figure 3:**
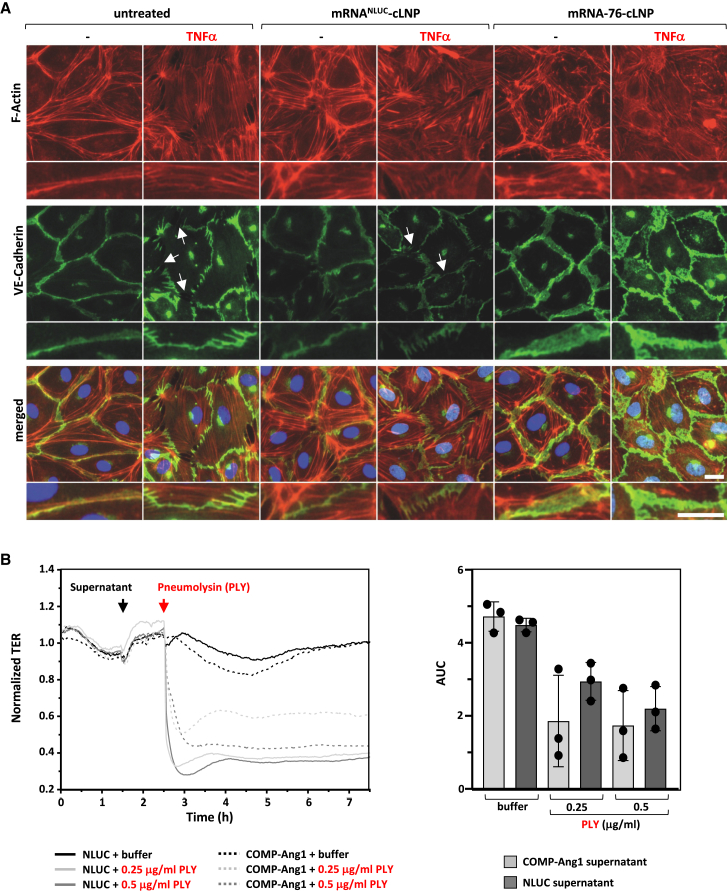
cLNP-mediated delivery of COMP-Ang1-encoding mRNA-76 stabilizes endothelial barrier *in vitro* (A) Effect of COMP-Ang1 on F-actin remodeling and VE-cadherin distribution in isolated HPMECs. HPMECs grown on glass coverslips were transfected for 2 h using mRNA-cLNP formulations with control mRNA^NLUC^ or with mRNA-76 in serum-free media; cells were then kept in serum-containing media for a further 16 h before addition of TNF-α (10 ng/mL) for another 6 h followed by immunofluorescence staining with VE-cadherin-specific antibody and with Alexa Fluor 594 phalloidin to detect actin filaments (F-actin). Arrows indicate disrupted VE-Cadherin complexes. (B) TER measurements of HPMEC monolayers. HPMEC were incubated for 60 min with supernatant from mRNA-76 (encoding COMP-Ang1) or mRNA^NLUC^ (Nano-Luciferase) transfected HeLa cells and stimulated with the indicated amount of PLY (0.25, 0.5 μg/mL). PLY stimulation decreased TER of HPMEC monolayers, displaying loss of endothelial integrity when incubated with control luciferase supernatant (solid curves). Preincubation with COMP-Ang1 containing supernatant attenuated the PLY-induced TER decrease (dashed curves). After PLY stimulation the AUC was calculated and confirmed the observation that the “COMP-Ang1 supernatant” reduces the PLY-induced endothelial barrier failure. Data are represented as mean ± SEM, n = 3 per group.

Regulation of endothelial barrier stabilization by Ang1/Tie2 signaling has been shown to be mediated by vascular endothelial (VE)-cadherin–dependent cell-cell adhesion and cortical actin formation, since the cytoplasmatic tail of VE-cadherin is known to contain several phosphorylation sites with different distinctive and selective effects on EC function.^28^ However, inflammatory conditions lead to the disruption of VE-cadherin complexes by phosphorylation-dependent internalization and by degradation as well as to the formation of actin stress fibers. We analyzed VE-cadherin distribution in HPMEC cells upon tumor necrosis factor alpha (TNF-α) challenge by immunofluorescence staining and by microscopic analysis. As reported previously, TNF-α was shown to induce the disruption of cell-cell adhesion, as verified by disruption intercellular VE-cadherin staining (Figure 3A, arrows), and by the formation of actin stress fibers in untransfected cells as well as in cells transfected with control mRNA^NLUC^-cLNP. However, the disruption of VE-cadherin staining as well as stress fiber formation was prevented in the presence of TNF-α in cells transfected with mRNA-76-cLNP. Moreover, COMP-Ang1 expressing HPMEC displayed a broader intercellular VE-cadherin staining pattern compared to the rather thin intercellular staining in untreated cells, even in the presence of TNF-α. This observation argues for the existence of an increased VE-cadherin trans-interaction area at overlapping cell edges, as previously described by Birukova et al.^29^ Following the demonstration of functional Tie2 activation on VE-cadherin complex stabilization, we investigated the potential effect of mRNA-76-directed expression of COMP-Ang1 on pneumolysin (PLY)-evoked barrier failure in cell culture (Figure 3B). Recovery of the PLY-evoked barrier failure was measured by monitoring transcellular electrical resistance (TER).^30^ TER measurements are performed by applying an alternating current electrical signal across electrodes placed on both sides of a cellular monolayer and subsequently measuring voltage and current to calculate the electrical resistance of the respective barrier.^31^ A significantly improved transcellular electrical resistance was observed with the mRNA-76 conditional medium at the lowest PLY concentration of 0.25 μg/mL, but not with the supernatant from HeLa cells transfected with luciferase mRNA. This effect was observed in PLY-induced barrier failure experiments after pretreatment with conditional supernatant for 60 min. These data argue for an underlying mechanism by which mRNA-76 treatment leads to the expression and secretion of functional COMP-Ang1 in a paracrine manner on microvascular EC (HPMEC) monolayers as demonstrated by its ability to attenuate the PLY-induced TER decrease. These findings confirm the proposed mode of action of our mRNA-76-cLNP-expressing COMP-Ang1 in diminishing inflammation-induced hyperpermeability via its respective activation of the Tie2 signaling pathway.

### Localized expression of COMP-Ang1 in murine lungs by cLNP-mediated lung delivery of mRNA-76

**Figure 4:**
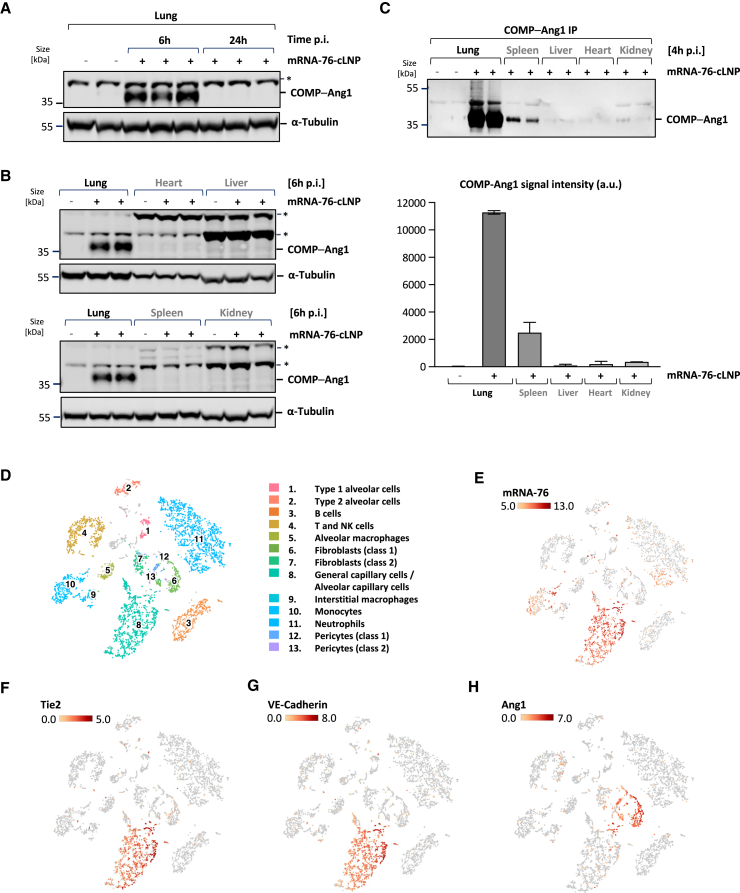
Localized expression of COMP-Ang1 in murine pulmonary ECs by cLNP-mediated delivery of mRNA-76 (A) Immunoblot of lung tissue lysates from individual animals 6 and 24 h after administration of 1.5 mg/kg mRNA-76-cLNP (+) or saline control (−). (B) Immunoblot of different tissue lysates from individual animals 6 h postadministration of 1.5 mg/kg mRNA-76-cLNP (+) or saline control (−). (C) Immunoprecipitation (IP) (top) and signal quantification (bottom) of COMP-Ang1 protein using Tie2-Fc-coupled magnetic beads from indicated tissue samples of individual animals 4 h postadministration of 1.5 mg mRNA-76-cLNP (+) or saline control (−). Quantification data are represented as mean ± SEM. (D) Loupe-based t-SNE clustering of murine lung cell types clustered by single-cell transcriptional analysis, showing a total of 13 distinct cell-type clusters as indicated by numbers. (E) t-SNE plots showing levels for COMP-Ang1-encoding mRNA-76. (F, G) t-SNE plots showing levels for canonical markers of ECs Tie2 (F) and VE-cadherin (G). (H) Cells expressing Ang1 (pericytes class 1 and 2, fibroblasts class 1). The intensity of expression (Log2) is indicated by red coloring. ∗, unspecific band. NK, natural killer; p.i., post injectionem.

Expression of mRNA-76-directed COMP-Ang1 *in vivo* was analyzed by immunoblot with an Ang1-specific antibody on lung protein-lysates from mice treated with 2 mg/kg mRNA-76-cLNP by intravenous tail vein administration. Robust COMP-Ang1 expression was detected in lung lysates from mice 6 h after treatment, declining over time (data not shown) with no expression levels observed in mice 24 h posttreatment due to the transient nature of mRNA expression (Figure 4A). As expected from previous studies with cLNPs, mRNA-76-cLNP-mediated robust lung tissue–selective COMP-Ang1 expression, while generating almost no COMP-Ang1 expression in tissue lysates from heart, liver, and kidney, with minor levels in spleen (Figure 4B). Immunoprecipitation was performed on different organ lysates using Tie2-Fc fusion protein to capture COMP-Ang1 to confirm lung-selective COMP-Ang1 expression. Even with this more sensitive detection method, the predominant COMP-Ang1 expression was observed in lung lysates, with some minor expression also seen in the spleen (Figure 4C). No significant expression of COMP-Ang1 was detected in liver, heart, and kidney, thus confirming the selective pulmonary delivery of mRNA-76-cLNP.

We performed single-cell RNA sequencing on cells derived from murine lungs to characterize the specific cell types with mRNA-76 uptake. Lungs from two mice treated with 1.5 mg/kg mRNA-76-cLNP (2 h postadministration) were obtained, and lung cell suspension was subjected to droplet-based single-cell gene expression profiling (see experimental workflow in Figure 1E). We obtained 5,100 high-quality transcriptome readings and were able to identify 13 cell clusters with main cell types of the respective lung samples by using dimensionality reduction via t-SNE and graph-based clustering (Figure 4D). We observed mRNA-76 reads to be largely restricted to cluster 8 and cluster 10, representing cells of the alveolar capillary endothelium (gCap, general capillary cells) and aCap (alveolar capillary cells) recently termed aerocytes, as well as to monocytes, respectively (Figure 4E).^32^ Minor amounts of mRNA-76 appeared to also be present in a subcluster of the pulmonary neutrophils (cluster 11). We analyzed the mRNA reads of Tie2 and VE-cadherin, both EC-specific expressed genes, for the confirmation of endothelial-specific clustering for mRNA-76 into cluster 8. The overlap with the reads for both markers confirmed cell type specificity of mRNA-76 delivery (Figures 4F and 4G). In contrast to mRNA-76, endogenous Ang1 mRNA was mainly detected in cluster 6, which does not overlap with reads for the Tie2 receptor mRNA (Figure 4E). The observed cell type–specific expression profiles suggest a paracrine Tie2 activation for the endogenous Ang1 (Figure 4H), as well as a potential autocrine Tie2 activation by ectopically expressed, mRNA-76-encoded COMP-Ang1.

### mRNA-76-mediated Tie2 pathway activation in murine lungs

**Figure 5:**
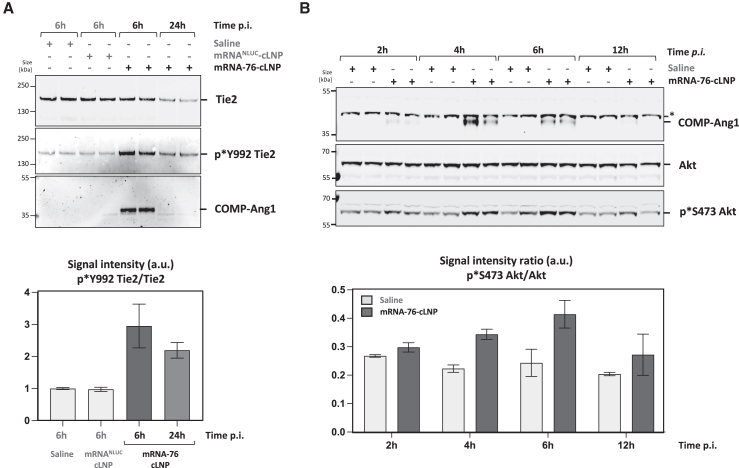
cLNP-mediated delivery of mRNA-76 leads to time-dependent Tie2 pathway activation *in vivo* (A) Immunoblot (top) and signal quantification (bottom) after IP of murine Tie2 from lung samples from individual animals analyzed for Tie2 protein levels, Tie2 phosphorylation, and levels of coimmunoprecipitated COMP-Ang1 at indicated time points after i.v. administration of 1.5 mg/kg mRNA-76-cLNP. Quantification data are represented as mean ± SEM. (B) Immunoblot (top) and signal quantification (bottom) of murine lunge tissue lysates from individual animals analyzing COMP-Ang1 expression level and Akt phosphorylation status at indicated time points after i.v. administration of 1.5 mg/kg mRNA-76-cLNP or saline. Quantification data are represented as mean ± SEM. ∗, unspecific band.

We then demonstrated Tie2 pathway activation in lung tissue to be induced by mRNA-76 COMP-Ang1 expression in murine lung tissue. Activation of phosphatidylinositol 3-kinase signaling including Akt phosphorylation is known to be a downstream effect of Tie2 receptor activation. Of note, demonstrating COMP-Ang1-mediated activation of Tie2 signaling is difficult due to the low levels of Tie2 protein in ECs within total lung lysates in healthy lungs. Therefore, Tie2 was immunoprecipitated from lung tissue lysates of mice previously treated with mRNA-76 using Tie2-specific antibodies. Tie2 phosphorylation of the lung was subsequently shown by immunoblot analysis of immunoprecipitated samples using anti-phospho-Tie2 antibody (p∗Y992) (Figure 5A), lasting at least 24 h after a single treatment. Signal quantification of phosphorylated Tie2 was measured relative to corresponding total Tie2 levels. In addition, immunoblot analysis using Ang1-specific antibodies showed coimmunoprecipitation (IP) of COMP-Ang1, thus demonstrating efficient binding of human COMP-Ang1 to murine Tie2. Since Akt phosphorylation upon COMP-Ang1 stimulation is mediated by Tie2 activation, whole-lung tissue lysates from mice treated with 1.5 mg/kg mRNA-76-cLNP were analyzed for COMP-Ang-1 expression, Akt expression, and Akt-phosphorylation by immunoblot (Figure 5B) in comparison to control lysates. Akt phosphorylation levels were shown to be highest 6 h post-mRNA-76-cLNP treatment.

### mRNA-76 treatment reduces PLY-evoked vascular permeability in an isolated perfused mouse lung (IPML) model for vascular leakage

**Figure 6:**
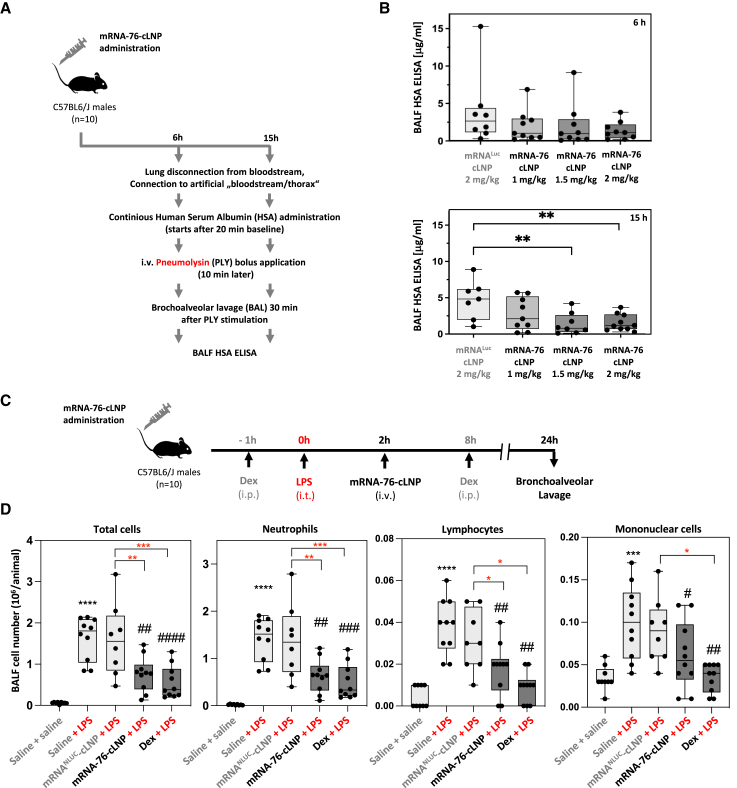
Systemic mRNA-76 treatment stabilized endothelial barrier lung function *in vivo* (A) Study outline of *ex vivo* perfused and ventilated mouse lung model. At 6 and 15 h postadministration of mRNA-76-cLNP (1, 1.5, and 2 mg/kg) or control mRNA^FLUC^-cLNP (2 mg/kg) lungs were isolated and stimulated with PLY (1.4 μg/mL) for 1 min. After 30 min, lung vascular permeability was assessed by quantifying respective concentrations of continuously infused HSA in the BALF. (B) Treatment with mRNA-76 significantly decreases PLY-induced hyperpermeability of mouse lungs as compared to treatment with control mRNA^FLUC^-cLNP 6 h (top) and 15 h (bottom) as shown by HSA ELISA. Quantification data are represented as mean ± SEM (n = 10, ∗∗p < 0.01 between indicated groups) concentration of HSA in BALF. HSA concentration of individual mice are indicated as dots. (C) Systemic mRNA-76 treatment prevents neutrophilia transmigration in LPS-induced pulmonary inflammation *in vivo*. Study outline: animals (n = 10) were challenged intratracheally with 0.9 % w/v saline or LPS (3 mg/kg). A fixed volume of 50 μL, equal to an approximate intratracheal dose volume of 2.5 mL/kg, based on a 20 g mouse, was applied intratracheally. 2 h after LPS administration, mRNA-76-cLNP (1.5 mg/kg i.v.) or mRNA^NLUC^-cLNP (1.5 mg/kg i.v.) was administered i.v., and BALF was analyzed by flow cytometry after 24 h. The assay control group was treated with dexamethasone (Dex, 3 mg/kg, i.p.) at 1 h prior and 8 h after LPS treatment. (D) Effect of COMP-Ang1 expression on BALF total and differential cell counts of indicated cells in a murine model of LPS-induced pulmonary inflammation. Quantification data are represented as mean ± SEM. Black asterisks ∗∗∗p < 0.001, ∗∗∗∗p < 0.0001 when compared to saline-challenged control group; red asterisks ∗p < 0.05, ∗∗p < 0.01, ∗∗∗p < 0.001 compared to LPS challenged mRNA^NLUC^-cLNP group. #p < 0.05, ##p < 0.01, ###p < 0.001, ####p < 0.0001 as compared to LPS-challenged and vehicle-treated groups.

A PLY-stimulation experiment was performed on isolated perfused mouse lungs (IPML model) to demonstrate mRNA-76 efficacy in preventing and/or decreasing lung hyperpermeability by inflammation (Figure 6A). In our IPML model, PLY stimulation increased lung permeability 30 min after application, as described previously.^33^^,^^34^ Here, treatment with mRNA-76 was shown to significantly decrease PLY-induced hyperpermeability of mouse lungs as compared to the luciferase controls (Figure 6B). A dose-dependent reduction of levels of the previously administered human serum albumin (HSA) in the bronchoalveolar lavage fluid (BALF) was observed 6 and 15 h post-mRNA-76 treatment as compared to respective levels in the control experiments, this observation being indicative of a reduction of vascular leakage by the expression of mRNA-76. However, under these *in vivo* assay conditions, statistical significance was observed only at 15 h (Figure 6B). These results suggest that mRNA-76 pretreatment is effective in preventing PLY-induced vascular leakage in the IPML model, and respective observations are consistent with the previously established mode of action of Tie2 activation by mRNA-76-directed expression of COMP-Ang1 in lung tissues. The effective dose in this mouse efficacy model is approximately 1 mg/kg for the 6 h time point and marginally higher at later time points (15 h). This decreasing effect on vascular leakage at later time points with lower doses suggests a more sustained expression with higher mRNA-76 dosing.

### mRNA-76-directed COMP-Ang1 expression reduces neutrophil influx after lipopolysaccharide (LPS) challenge in murine lungs

We observed a significant therapeutic effect on endotoxin/LPS-induced neutrophilia after temporal and spatial expression of COMP-Ang1 in a murine model of endotoxin (*Escherichia coli* LPS-induced pulmonary inflammation (Figure 6C). Single mRNA-76-cLNP treatment in our experimental model resulted in a significant decrease in LPS-induced neutrophil influx into the alveoli (Figure 6D). Levels of the decreased neutrophil influx effect were in the same range as those of prophylactic and therapeutic dexamethasone treatments used as positive controls in the assay. No significant reduction regarding cell influx was observed with mRNA^NLUC^-cLNP compared to vehicle control mice. Also, no changes in LPS-induced vascular leakage (e.g., changes in wet lung weights) were observed with mRNA-76-cLNP treatment compared to control mRNA formulation, indicating that vascular leakage and leukocyte emigration do not necessarily occur together in the blood vessels of the lung. The fact that elevated levels of circulating cytokines are not perturbed by the enhancement of vascular integrity and hence the notion that leakage can be regulated distinctly from the inflammatory response has been discussed previously by others.^35^ In general, the effect of mRNA-76-cLNP treatment in rodent models for LPS-induced lung injury could also be dependent on the time point at which samples are obtained and at which respective physiological measurements are taken. The LPS model may therefore not be an adequate means by which to simultaneously address the question of both neutrophil efflux and lung edema at similar time points 24 h postendotoxin challenge. This is in contrast to the IPML model used primarily for experimentally demonstrating reduced vascular leakage. In our murine model of LPS-induced pulmonary inflammation, intravenous mRNA-76-cLNP treatment showed a strong therapeutic effect in reducing leukocyte extravasation and/or transmigration of neutrophils for the time point(s) analyzed. Leukocyte influx reduction and reduction in edema formation is highly desirable to counteract tissue injury.

Taken together, our experiments in the IPML mouse model confirm a dose-dependent therapeutic effect of COMP-Ang1 on endothelial permeability, as well as a therapeutic efficacy on dysregulated lung inflammation in the LPS-induced pulmonary neutrophilia mouse model. Underlying pathophysiological mechanism, loss of endothelial permeability, and dysregulated lung inflammation are hallmark events of early acute respiratory distress syndrome (ARDS) syndrome.

## Discussion

One of the major challenges in the development of mRNA-based therapeutics continues to be the safe and efficacious systemic delivery of mRNA to specific organs and cells *in vivo*. The long-standing problem has been that targeting of systemically administered, mRNA-coformulated LNPs has largely been confined to the liver and, albeit to a much lesser degree, to the spleen (most likely by splenic macrophages phagocytosing nondelivered particles due to its natural function as part of the reticuloendothelial system).^36^ Neutral LNPs (nLNPs) exhibit a distinct targeting profile and are known to preferentially target the liver, this being due to their interaction with apolipoprotein E (ApoE) in blood and subsequent ApoE-low-density lipoprotein receptor-mediated endocytosis in hepatocytes.^37^

In our experiments, we used a refined second-generation cLNP system, building upon our previous work with siRNA-lipoplex formulations. This earlier research effectively showed that first-generation siRNA-lipoplexes, with a lipid composition similar to the present study, can induce RNA interference in mouse vasculature across various organs, including the lung, heart, and liver.^5^^,^^6^ However, the physicochemical properties of the new cLNP generation under discussion, especially in terms of particle size, size distribution, zeta potential, and particle morphology, have seen a significant transformation. This change is credited to the integration of a certain pH buffer system in the complexation process and the use of advanced microfluidic mixing techniques (Figures 1A–1C). As a result, these new cLNPs demonstrate a unique propensity for specifically targeting the lung vasculature due to their certain particle size, surface properties, and the unique anatomy of the lung vasculature.^38^^,^^39^ The exact mechanism for this phenomenon is still under investigation but can already be attributed in part to the specific electrostatic interaction with the negatively charged glycocalyx layer on the surface of the lung vasculature. Moreover, the endothelium of the lung expresses a range of receptors, including scavenger receptors and integrins, which can mediate the uptake of cationic nanoparticles decorated with serum proteins (“corona formation”).^11^^,^^13^ The complex and diverse architecture of the lung vasculature may also contribute to the observed, highly cell-type–specific targeting of the applied cLNPs (Figures 1F, 4D, and 4E), since the dense network of lung capillaries in the alveoli combine a large surface with a very thin barrier for the efficient exchange of oxygen and carbon dioxide between air and blood, thus maximizing the surface area available for interaction with nanoparticles.^32^

Here, we report for the first time a novel cLNP nucleic acid delivery system for transporting mRNAs selectively and almost exclusively into capillary ECs of the lung vasculature. Furthermore, we demonstrate treatment with the respective mRNA-76-cLNP to achieve transient and localized expression of functional COMP-Ang1 in the lung vasculature (Figures 4 and 5). We show a dose-dependent therapeutic effect of mRNA-76-cLNP on lung endothelial permeability in an IPML mouse model, as well as its respective therapeutic efficacy on dysregulated lung inflammation (Figure 6). Both pathophysiological mechanisms, namely increased endothelial permeability and dysregulated lung inflammation, are also hallmark events at the onset of early/mild ARDS.^15^^,^^40^ To date there is no medication available for treatment and for the further reduction of ARDS mortality in patients. Hence, the advantage of our novel mRNA-76-cLNP compound as described above in the context of ARDS is its ability to be delivered and to act in a highly spatially restricted manner, enabling the Tie2 agonist to rapidly and almost exclusively target the specific site of the endothelial capillary bed where the pathophysiological lung edema occurs. Our new approach and technology thus overcome the limitations previously observed of efficacious and tissue-targeted administration of recombinant multimeric proteins for activating Tie2 signaling. Tie2 activation has been previously reported to require a specific distance of the Ang1-binding domains in relation to the two Tie2 monomers.^41^ This very specific steric–spatial interaction is potentially facilitated by the pentameric COMP-Ang1 as compared to the dimeric CMP-Ang1 in low Tie2-expressing HPMEC cells. As a result, the observed higher and robust COMP-Ang1-mediated Tie2 activation in our experiments is most likely caused by its higher solubility compared to WT-Ang1, as well as by its more efficient multimerization into active pentamers as compared to that of CMP-Ang1. The difference in Tie2 activation (Figure 2F) in our hands led us to concentrate on mRNA-76 encoding COMP-Ang1 for our further experiments. Subsequently, we demonstrated mRNA-76-mediated COMP-Ang1 expression and Tie2 activation to enhance intercellular cell adhesion by way of monitoring transcellular electrical resistance in HPMEC monolayers (Figure 3B). The observed stabilization of endothelial barrier function in *in vitro* cell culture (Figure 3A) was consistent with our *in vivo* results, demonstrating a reduction of PLY-evoked permeability in isolated perfused and ventilated mouse lungs after mRNA-76 treatment (Figures 6A and 6B).

We did not observe changes in LPS-induced edema formation (e.g., changes in wet lung weights) with mRNA-76-cLNP compared to control mRNA formulation in this model (Figures 4D and 4E). Our data suggest that vascular leakage and leukocyte extravasation do not necessarily occur together in blood vessels of the lung, which has been discussed previously by others.^35^ In general, these findings in murine models for LPS-induced lung inflammation may also depend on the time point at which samples are obtained and at which the corresponding physiological data are captured. However, we observed a dose-dependent response in the IPML model addressing vascular leakage at two different time points (Figure 6). In addition, intravenous mRNA-76-cLNP treatment was shown to intervene with leukocyte and in particular neutrophil extravasation into the alveoli after LPS challenge. It should be noted, however, that the reduction of the influx of leukocytes in addition to a reduction in edema formation may be clinically highly desirable.^42^ Within that context, many studies have suggested that neutrophil recruitment to the lungs is associated with disease severity during ARDS development, and neutrophils have been implicated as drivers of disease pathogenesis.^15^^,^^42^ Taken together, the data in our IPML mouse model demonstrate a dose-dependent therapeutic effect of mRNA-76-cLNP regarding aberrant endothelial permeability, whereas the LPS-induced pulmonary neutrophilia mouse model depicted in Figure 6B shows therapeutic efficacy of our novel cLNP delivery system with its mRNA-76 cargo/payload on ameliorating dysregulated lung inflammation.

Thus, the advantage of our novel mRNA-76-cLNP compound is its ability to act in a highly spatially restricted manner, targeting almost solely the exact site of lung edema and/or injury. This strong spatial restriction of action of our mRNA compound permits more rapid onset of Tie2 pathway activation, as well as improved pharmacodynamics compared to those delivery modalities requiring continuous treatment owing to the respective rapid clearance of recombinant proteins from the systemic circulation. With the understanding that the angiopoietin/Tie (Ang/Tie) family has an established role in vascular physiology in regulating angiogenesis, vascular permeability, and inflammatory responses, our data corroborate effectiveness and mode of action of the clinical approach of treating dysfunction of the lung vascular endothelium (lung edema) in ARDS patients with the transient expression of a therapeutic COMP-Ang1 mRNA-cLNP modality.

## Materials and methods

### mRNA synthesis

Codon-optimized mRNAs containing 5′ UTR sequence and signal peptide sequence of human MCP1 (NM_002982.3), 3′ UTR sequence of human von Willebrand factor (NM_000552.4), and coding regions of human Ang1 (mRNA-52), COMP-Ang1 (mRNA-76), CMP-Ang1 (mRNA-59), Tie2 (mRNA^Tie2^), Firefly luciferase (mRNA^FLUC^), and secreted NanoLuc (mRNA^NLUC^) proteins were synthesized and purified by BioSpring GmbH (Frankfurt am Main, Germany) and AmpTec (now Merck, Hamburg, Germany). Uridine was globally replaced with N1-methylpseudouridine.

### LNP preparation and characterization

The cationic lipid (l-arginyl)- l-2,3-diamino propionic acid-*N*-palmityl-*N*-oleyl-amide (Pantherna Therapeutics, Hennigsdorf, Germany), the helper lipid 1,2-diphytanoyl-*sn*-glycero-3-phosphoethanolamine (Corden Pharma, Plankstadt, Germany), and the PEGylated lipid 1,2-distearoyl-*sn*-glycero-3-phosphoethanolamine-*N*-[methoxy(polyethylene glycol)-2000] (ammonium salt) (Avanti Polar Lipids, Alabaster, AL) were dissolved in pure ethanol with a molar ratio of 50:49:1, mixed with mRNA dissolved in 10 mM sodium citrate buffer at pH 5.5, resulting in a lipid:mRNA mass ratio of m:m = 20, with an mRNA concentration of 100–280 μg/mL in the final formulation. Specifically, the aqueous mRNA solutions and the ethanolic lipid mixtures were combined at a volume ratio of 2:1 (aqueous:ethanol) and flow rates of 18 mL/min using a microfluidic mixer (NanoAssemblr; Precision Nanosystems, Vancouver, BC, Canada). Formulations were dialyzed against 10 mM Tris (pH 7.4) with 9% sucrose in Slide-A-Lyzer dialysis cassettes (MWCO 3.5k, Thermo Scientific, Rockford, IL) for at least 18 h at 4°C. The formulations were tested for particle size and zeta potential (Zetasizer Nano ZS, Malvern Instruments, Malvern, UK) as well as for RNA encapsulation and for total RNA content (Quant-iT RiboGreen RNA Assay Kit, Thermo Fisher Scientific, Waltham, MA). The formulations were found to have a Z-average between 80 and 90 nm, a polydispersity index <0.1, and a zeta potential between +5 and −10 mV using 10 mM Tris (pH 7.4) with 9% sucrose as the suspension buffer. The mRNA encapsulation was always >95%. The formulations were stored at −80°C until further use.

### Single-cell RNA sequencing

Single-cell suspensions generated from fresh mouse lung samples were loaded onto the chip G (10x Genomics, Pleasanton, CA) and processed according to the Chromium Next GEM Single Cell 3′ workflow using the Chromium Controller device (10x Genomics). For each sample, 11,000 single cells were loaded, with an average cell viability of 88%. The resulting libraries were sequenced using a NextSeq2000 device (Illumina, San Diego, CA). For the analysis of the sequencing data, a new reference was constructed based on the GRCm39 genome and the GENCODE M29 annotation, which included the mRNA-76 transgene contigs along with their annotation and was then filtered according to the standard recommendations of 10x CellRanger (version 7.0.0) and packaged for use by the ‘cellranger mkref’ command using default parameters. All of the sequencing runs were demultiplexed with ‘cellranger mkfastq’ using default parameters. The demultiplexed samples were processed with ‘cellranger count’ using the nondefault parameter ‘--expect-cells = 5000’.^43^^,^^44^

### Transcellular electrical resistance of human pulmonary microvascular ECs

HeLa cells were first treated for 24 h with LNP-formulated mRNA-76 or with a luciferase mRNA (see above) to generate conditional serum-free DMEM medium containing secreted COMP-Ang1 or secreted luciferase. Moreover, HPMEC were grown to confluency on evaporated gold microelectrodes (8-well array with 10 electrodes per well, (ibidi GmbH, Gräfelfing, Germany, catalog no. 72010) and connected to an electrical cell-substrate impedance sensing system (Applied Biophysics, Troy, NY)^16^^,^^17^ to enable continuous monitoring of TER. After monitoring baseline readings for 60–90 min, HPMEC medium was mixed 1:10 with the conditional HeLa media (COMP-Ang1 supernatant or Luc [luciferase] supernatant) for 30 or 60 min before treatment with three different concentrations of PLY (0.25, 0.5, and 1.0 μg/mL) to evoke a barrier failure in HPMEC. TER values from each microelectrode were continuously monitored for 5 h after PLY stimulation and normalized as the ratio of measured resistance to baseline resistance. The area under the curve (AUC) was calculated from start of PLY stimulation on.

### IPML experiments

Intravenous tail vein administration with either mRNA^LUC^ (2 mg/kg) or mRNA-76 (2, 1.5, or 1 mg/kg) was performed 6 or 15 h before the experiment in the isolated mouse lung model was started. Mice were anesthetized, placed in a 37°C heated chamber, tracheotomized, and ventilated as described.^34^ After laparotomy, final blood collection via the vena cava, sternotomy, and cannulation (pulmonary artery, left atrium), lungs were perfused with Krebs-Henseleit-hydroxyethylamylopectine buffer (Serag-Wiessner GmbH, Naila, Germany) supplemented with sodium bicarbonate. Lungs were ventilated by negative pressure and perfused for 20 min to establish baseline conditions. Then, 0.04% HSA (CSL Behring, Marburg, Germany; human albumin 20% Behring) was admixed continuously to the perfusate 10 min before i.v. bolus application of recombinant PLY (1.4 μg/mL). Thirty minutes after PLY challenge, BAL was performed, and the concentration of HSA was measured in BAL fluid via ELISA (Bethyl Laboratories, Montgomery, TX).

### Neutrophilia transmigration experiment

Animals (n = 10) were challenged intratracheally with 0.9% w/v saline or with LPS (3 mg/kg). Two hours later, animals were treated with mRNA-76-cLNP or with control mRNA^LUC^-cLNP using a dosage of 1.5 mg/kg via i.v. tail vein injection. Control group animals received intraperitoneal (i.p.) injections of dexamethasone (3 mg/kg) 1 h before and 8 h after LPS administration. Animals were sacrificed after 24 h and the airway lavaged with 0.3 mL of PBS using an inserted cannula. BAL from each mouse was centrifuged and the cell pellet was resuspended in 0.5 mL PBS. A total and differential cell count of the BALF supernatant was performed using the XT-2000iV (Sysmex, Milton Keynes, UK). The samples were vortexed for approximately 5 s and analyzed. Total and differential cell counts (including neutrophils, lymphocytes, and mononuclear cells [includes monocytes and macrophages]) were measured as the number of cells per animal.

### Cell transfection *in vitro*

HeLa cells were transfected with mRNAs using Lipofectamine MessengerMax (Invitrogen, Waltham, MA, catalog no. LMRNA001) according to the manufacturer’s protocol. Briefly, cells were seeded in 150 cm^2^ culture flasks (TPP, Trasadingen, Switzerland, catalog no. 90151) at 50% confluency in serum-free DMEM (Gibco, Grand Island, NY, catalog no. 61965–026). A total of 15 h later, cells were transfected for 2 h with indicated amounts of MessengerMax-formulated mRNAs according to the manufacturer’s protocol. The transfection media was removed and replaced with fresh serum-free DMEM for the indicated time points. After 24 h, the supernatant was removed (approximately 25 mL) and 10-fold concentrated using Amicon 3 kDa spin columns (Merck, catalog no. UFC900308) and then brought to a final volume of 2 mL for subsequent Western blot analysis (Figures 1B and 1C) or directly used for Tie2 activation studies (Figures 2A, 2B, and 3B). HPMECs (Promocell, Heidelberg, Germany, catalog no. C-12281) were seeded onto 6-well plates (Thermo Scientific, catalog no. 140675) and left for 3 days until 100% confluency in full supplemented ECM MV2 cell media (Promocell, catalog no. C-22022). Fifteen hours before, transfection cells were placed in serum-reduced ECM MV2 media (containing 10% of original cell growth supplement MV2). HPMECs were transfected with preformulated mRNA-cLNP at the indicated concentrations in serum-free ECM media for 2 h. After transfection, media was replaced with serum-reduced ECM MV2 and left for the indicated time points.

### Western blot analysis

Cells were washed with ice-cold PBS twice and lysed with ice-cold radioimmunoprecipitation assay (RIPA) buffer (Thermo Scientific, catalog no. 89901) supplemented with Halt protease and phosphatase inhibitor cocktail (Thermo Scientific, catalog no. 78441). Tissue samples were solubilized in T-PER tissue protein extraction rReagent (Thermo Scientific, catalog no. 78510) supplemented with Pierce protease and phosphatase inhibitor tablets (Thermo Scientific, catalog no. A32958) using Qiagen TissueLyser LT (Qiagen, Hilden, Germany, catalog no. 69980). A mixture of cell or tissue lysate, 50 mM dithiothreitol NuPAGE reducing agent (Invitrogen, catalog no. NP0009) and NuPAGE sample buffer (Invitrogen, catalog no. NP0007) was heated at 70°C for 10 min, electrophoresed in NuPAGE 4%–12% Novex Bis-Tris gels and 3-(*N*-morpholino)propanesulfonic acid buffer (all from Invitrogen, catalog nos. NP0335BOX, NP0001) and transferred to nitrocellulose membrane (Cytiva, Marlborough, MA, catalog no. 10600003; NuPAGE transfer buffer (Invitrogen, catalog no. NP00061). After a 60-min blocking step (Intercept blocking buffer; LI-COR, Ponoka, AB, Canada, catalog no. 927–60001) membranes were washed with Tris-buffered saline with Tween 20 (Cell Signaling Technology, Danvers, MA, catalog no. 9997S) and probed at 4°C overnight with specific primary antibodies diluted in Intercept Antibody Diluent (LI-COR, catalog no. 927–65001). Binding of primary antibodies was detected by near-infrared IRDye secondary antibodies (LI-COR, catalog nos. 926–32213, 926–32214, 926–68072) and near-infrared signals were analyzed using LI-COR Odysee CLx. Band intensities were analyzed using Empiria Studio Software 2.1.0134 (LI-COR). The primary antibodies used were angiopoeitin 1 (Abcam, Cambridge, UK, catalog no. ab183701), human Tie2 (Cell Signaling Technology, catalog no. 7403), mouse Tie2 (Merck, catalog no. 05–584), pY992 Tie2 (R&D Systems, Minneapolis, MN, catalog no. AF2720), Akt (Cell Signaling Technology, catalog nos. 9272 and 2920S), pS473 Akt (Cell Signaling Technology, catalog no. 4060), S6K (Cell Signaling Technology, catalog no. 9202), pT389 S6K (Cell Signaling Technology, catalog no. 9234), FoxO1 (Cell Signaling Technology, catalog no. 2880), pT389 FoxO1 (Cell Signaling Technology, catalog no. 9461).

### IP

Anti-Tie2 antibody ab33 (R&D Systems, Merck, catalog no. 05–584) for Tie2 IP or Tie2-Fc fusion protein (R&D Systems, catalog no. 313-TI) for COMP-Ang1 IP were covalently coupled to M-270 Epoxy Dynabeads (Thermo Scientific, catalog no. 14311D) at a ratio of 15 μg protein per milligram of Dynabeads according to the manufacturer’s protocol. Briefly, 1.5 mg of antibody-coupled beads were incubated with tissue lysates overnight at 4°C. The beads were washed 5 times with 1 mL ice-cold RIPA buffer and finally denatured using 1× NUPAGE loading dye containing 1× NUPAGE reducing agent (all Thermo Scientific). The bound beads were removed by centrifugation and the lysates were analyzed by Western blotting analysis.

### Fluorescent microscopy

HPMECs were seeded in chamber slides (NUNC Lab-Tek, catalog no. 154534PK) at 1 × 10^5^ cells/mL in endothelial MV2 growth medium. Cells were incubated for 3–5 days at 37°C in 5% CO_2_. Cells were washed 2 times with prewarmed PBS (Thermo Scientific, catalog no. 14190), and the final PBS wash was replaced with endothelial MV2 basal medium devoid of serum and supplements. Formulations containing mRNA were diluted in basal medium and added to each well for a final concentration of 0.38 ng/well. Cells were returned to the incubator for an additional 2 h of incubation. Media containing formulations was removed and replaced with 10% MV2 growth medium for 16 h. For TNF-α treatment, 10% MV2 growth medium with TNF-α (R&D Systems, catalog no. 210-TA) or vehicle was spiked into each well for a final concentration of 10 ng/mL for a further 6 h. Cells were fixed in 4% paraformaldehyde (Thermo Scientific, catalog no. 15670799) for 10 min at room temperature (RT) and subsequently permeabilized in PBS containing 0.02% Triton X (PBS-T) for 2 × 10 min washes on a rocker at RT. The primary antibody mix was made up with rabbit anti-VE-cadherin (Cell Signaling Technology, catalog no. 2500) diluted at 1:400 in 2% donkey immunobuffer containing 0.02% Triton X, rocking at 4°C overnight. Primary antibodies were removed and washed in PBS-T 2 × 10 min on a rocker at RT. The secondary antibody mix was made up with donkey anti-rabbit (H + L) highly cross-adsorbed Alexa Fluor 647 (Thermo Scientific, catalog no. A-31573) diluted at 1:400 and Alexa Fluor 594 Phalloidin (Thermo Scientific, catalog no. A12381) at 1:2,000 in 2% donkey immunobuffer containing 0.02% Triton X rocking at RT for 5 h. Cells were washed in PBS-T for a further 2 × 10 min at RT. Nuclear staining was carried out with Hoechst (Tocris Bioscience, Bristol, UK, catalog no. 5117) diluted at 1:500 in PBS for 15 min, followed by a final PBS-T wash. Fluorescent images were acquired with a Nikon Eclipse Ti-U with an ELWD S Pan Fluor 40×/0.6 objective lens. Images were processed using ImageJ (NIH, Bethesda, MD).

### Animals

Female C57BL/6N mice (8–10 weeks, 18–20 g; Charles River, Sulzfeld, Germany) were used for all of the experiments. All of the animal experiments were approved by institutional and governmental German authorities (“Tierschutzbeauftragte” (animal welfare officer) and “Tierschutzausschuss” of Charité - Universitätsmedizin Berlin; and by the Landesamt für Gesundheit und Soziales Berlin; approval ID A0179/20) and were in accordance with the Federation of European Laboratory Animal Science Associations guidelines and recommendations for the care and use of laboratory animals (equivalent to American ARRIVE (Animal Research: Reporting of In Vivo Experiments)). Mice were randomly assigned to the appropriate experimental groups and were kept in closed, individually ventilated cages with filter hoods (type II-L, ZOONLAB GmbH, Castrop-Rauxel, Germany), under specific pathogen-free conditions, with free access to food and water, RT between 20°C and 22°C, air humidity between 50% and 65%, and 12-h light:dark cycle.

### Statistical analysis

For the LPS study (Figure 6D), a parametric analysis was performed if D’Agostino/Pearson and Shapiro-Wilk tests for variance homogeneity were not significant at the 1% level. Groups were compared using one-way ANOVA combined with Dunnet’s multiple comparison test. A nonparametric analysis was performed if normality tests were still significant at the 1% level. Groups were compared using nonparametric Kruskal-Wallis tests followed by Dunn’s posttest. For the IPML study, data were expressed as mean ± SEM. For the comparison of two groups, the Mann-Whitney *U* test was used when the data values were not normally distributed. The Student t test was used for the particle analysis of total internal reflection fluorescence microscopy data. Data analysis was performed using the Prism software (GraphPad Software, La Jolla, CA). For all of the statistical analyses, significant differences between the groups compared were expressed at the 5% (p < 0.05), 1% (p < 0.01), or 0.1% (p < 0.001) level.

## Data and code availability

The authors confirm that the data supporting the findings of this study are available from the corresponding author upon reasonable request. The scRNA sequencing datasets are deposited at GEO DataSets: GSE241514.
